# Responding to comments on “The possible pathogenesis of macular caldera in patients with North Carolina macular dystrophy.“

**DOI:** 10.1186/s12886-023-03099-6

**Published:** 2023-09-08

**Authors:** Zhe Zhu, Jun Xiao

**Affiliations:** 1 Medical Retina, Eye center of the second hospital of Jilin University, Jilin, China; 2 Room 304, 3rd Floor, Out patient Building, No.218, Ziqiang Street, Nanguan District, Changchun city, Jilin Province China

Thanks to Dr. Kent W. Small’s supplementary description of NCMD subtypes, here we only give a general description to lead to the content we hope to discuss next.

Dr. Small points out an error in our article, that is, “chromosome 16q16 needs to be corrected to 6q16”, which is important for readers to know. We also agree with Dr. Small’s opinions on and additions to the references. Since the main research perspective of this article was clinical and imaging findings, it was inevitable that there would be omissions about the genetics. We thank Dr. Small for his supplemental information that made our article more complete [1–15].

In the studies he provides, we do not find a description of a Chinese or Asian family with NCMD [14]. Therefore, we recommend some supplementary annotated information to give the reader any available information along those lines.

In our study, whole-genome sequencing (WGS) was not performed. Based on existing clinical features and imaging and genetic evidence, we consider the diagnosis of MCDR1 in this family to be reliable. Because of economic and technological constraints, most patients cannot undergo WGS or related professional genetic analyses in clinical settings. Therefore, it is still worthwhile to research and discuss economical but reliable clinical diagnostic criteria for NCMD. Given the current state of the art, it is true that WGS can provide patients with more valuable information, so we recommend that patients with applicable conditions undergo WGS whenever possible. This is also the focus of our next efforts [15, 16]. Regarding gene therapy, this article merely presents a potential concept. As Dr. Small has noted, the specific technical aspects still require further investigation.

We thank Dr. Small for providing additional references on PRDM13 and the diagnosis and misdiagnosis of NCMD. Additionally, we appreciate the notification to replace “Kent et al.” with “Small et al.” in some references [9, 10, 13, 17].

In the part on general clinical manifestations, the description of some family members with poor vision included the patient’s subjective feelings, and we also reported the patient’s specific visual acuity (VA). Through the descriptions in the article, it should not be difficult for readers to conclude that most NCMD patients have relatively good VA and can maintain their vision in the long term.

We agree with Dr. Small’s statement that macular abnormalities in NCMD are congenital macular hypoplasia/dysplasia, that is, a disease caused by abnormal embryonic development that exists at birth [12, 14, 15]. However, this term may not fully explain the specific pathogenic mechanisms underlying maculopathy in NCMD grade 3. Based on the imaging changes in this family, we speculate that during the macular development period of patients with grade 3 maculopathy, abnormalities in the retinal pigment epithelium (RPE) and choroid lead to neurosensory retinal degeneration. This process resembles atrophy; hence, the term ‘atrophy’ is employed to denote that process. Here, ‘atrophy’ and ‘hypoplasia/dysplasia’ are not mutually exclusive but rather complementary in nature. As research advances, we anticipate the emergence of more precise terminology to delineate the specific pathogenic processes of maculopathy in this disease.

Finally, thanks to Dr. Small for his valuable comments on the usage of the term “caldera” in our article [8].

## Data Availability

Not applicable.

## References

[1] Small KW, Weber JL, Roses AD, Pericak-Vance M (1992). North Carolina macular dystrophy maps to chromosome 6. Genomics.

[2] Small KW, Weber J, Pericak-Vance MA (1993). MCDR1 (North Carolina macular dystrophy) map to 6q14-q16. Ophthalmic Pediatr Genet.

[3] Rabb M, Small KW, Mullen L, Yelchits L, Udar N (1998). North Carolina macular dystrophy maps to the MCDR1 locus (MCDR1) phenotype in Central America. Am J Ophthalmol.

[4] Small KW, Puech B, Mullen L, Yelchits L (1997). North Carolina macular dystrophy phenotype in France maps to the MCDR1 locus. Mol Vis.

[5] Small KW, Mullen L, Yelchits S, Udar N, Kelsell R, Hunt D, Ronald Klein C, Garcia G, Gallardo, Weber B, Bernard Puech, Puech V, Saperstein D, Lim J, Haller J, Flaxel C, Kelsell R, Hunt D, Evans K, Lennon F, Pericak-Vance M (1999). North Carolina macular dystrophy (MCDR1) locus: a fine resolution genetic map and haplotype analysis. Mol Vis.

[6] Small KW, Garcia CA, Gallardo G, Yelchits S, Udar N (1998). North Carolina macular dystrophy in Texas. Retina.

[7] Small KW. North Carolina macular dystrophy. Trans Am Ophthalmological Soc XCVI: 926–61, 1998.

[8] Small KW, Agemy S, Shaya FS (2016). Terminology of MCDR1: what’s in a name?. JAMA Ophthalmol.

[9] Small KW, Tran EM, Small L, Rao RC, Shaya F (2019). Multimodal Imaging and Functional Testing in a North Carolina Macular Disease Family: Toxoplasmosis, Fovea Plana, and Torpedo Maculopathy are phenocopies. Ophthalmol Retina.

[10] Small KW, Vincent AL, Knapper CL, Shaya FS (2019). Congenital toxoplasmosis as one phenocopy of North Carolina Macular dystrophy (NCMD/MCDR1). Am J Ophthalmol Case Rep.

[11] Small KW, Van de Sompele S, Nuytemans K (2021). A novel duplication involving *PRDM13* in a turkish family supports its role in North Carolina macular dystrophy (NCMD/MCDR1). Mol Vis.

[12] Small KW, Wiggins R, Udar N, Silva-Garcia R, Avetisjan J, Vincent A, Shaya FS. North Carolina Macular Dystrophy (NCMD/MCDR1): Long-term follow-up of the original family. Ophthalmol Retina. 2022 Feb 10:S2468-6530(22)00062 – 8. doi: 10.1016/j.oret.2022.02.003. Epub ahead of print. PMID: 35151913. 10.1016/j.oret.2022.02.003 35151913

[13] Small KW, Jampol LM, Bakall B, Small L, Wiggins R, Agemy S, Udar N, Avetisjan J, Vincent A, Shaya FS. Best Vitelliform Macular Dystrophy (BVMD) is a phenocopy of North Carolina Macular Dystrophy (NCMD/MCDR1). Ophthalmic Genet. 2021 Dec 13:1–11. doi: 10.1080/13816810.2021.2010771. Epub ahead of print. PMID: 3489501. 10.1080/13816810.2021.2010771 34895015

[14] Small KW, DeLuca AP, Whitmore SS (2016). North Carolina Macular dystrophy is caused by Dysregulation of the retinal transcription factor PRDM13. Ophthalmology.

[15] Van de Sompele S, Small KW, Cicekdal MB (2022). Multi-omics approach dissects cis-regulatory mechanisms underlying North Carolina macular dystrophy, a retinal enhanceropathy. Am J Hum Genet.

[16] Wu S, Yuan Z, Sun Z (2022). A novel tandem duplication of PRDM13 in a chinese family with North Carolina macular dystrophy. Graefes Arch Clin Exp Ophthalmol.

[17] Watanabe S, Sanuki R, Sugita Y (2015). Prdm13 regulates subtype specification of retinal amacrine interneurons and modulates visual sensitivity. J Neurosci.

